# CORESS Feedback: Cases from the Confidential Reporting System for Surgery

**DOI:** 10.1308/rcsann.2026.0044

**Published:** 2026-05-01

**Authors:** HJ Corbett, W Hawkins

**Affiliations:** on behalf of the CORESS Advisory Board

## Abstract

CORESS is an independent charity. Past CORESS cases can now be identified using a searchable CORESS case database, available via our website (coress.org.uk). We are grateful to those who have provided the material for these vignettes, some of which come from NHS England and Getting It Right First Time reports. The online reporting form is available on the CORESS website, which also includes previous Feedback reports. Published cases are acknowledged by a Certificate of Contribution, which may be included in the contributor’s record of continuing professional development, or which may form part of appraisal or annual review of competence progression portfolio documentation. Contributions from surgeons in training are particularly welcome.

## Swab count troubles

### Case 330

A 20-year-old female patient with a childhood diagnosis of inflammatory bowel disease underwent a difficult re-do laparotomy for adhesive bowel obstruction. As was the surgeon’s usual practice, large swabs were placed around the prepped abdomen under the drapes. The case began in the afternoon but went on into the evening and a change of scrub staff took place mid-way through what was a very long operation with over six hours of surgical time. The swab count commenced as the surgeon began to close the abdomen but the mismatched count was not noted until the abdomen was closed and the drapes removed, at which time the swabs under the drapes could be counted. The scrub staff had assumed that there were five swabs under the drapes when in fact there were only four, which led to the realisation that there was a swab that was unaccounted for.

The abdomen was reopened and the fifth swab retrieved from the left upper quadrant. An apology was given to the patient and she recovered uneventfully.

### Reporter’s comments

The swabs placed under the drapes were mentioned during the scrub team handover but there was nothing written down to document how many had been used. A tired surgical team may have contributed to the events.

### CORESS comments

CORESS noted that it is not usual practice to place swabs from the count under the drapes, which was undoubtedly a contributing factor in this incident. Other key factors were the lack of documentation of the swabs on the white board and a change of staff during the procedure. Accountability for reconciliation of the swabs (the swab count) falls to the entire team and not just the surgeon or the scrub practitioner.

While on the one hand, the reconciliation process did work, the atypical nature of the swab placement was a critical factor. A regular situation report during a long case can provide the opportunity to update new team members on points discussed at the briefing or (as in this case) during the operation.

The Health Services Safety Investigations Body (HSSIB) has recently updated its investigation report, which highlights the complexity of the process, and makes recommendations in collaboration with the Centre for Perioperative Care and the Association for Perioperative Practice. The report can be found on the HSSIB website.

Reference1.Health Services Safety Investigations Body. Retained swabs following invasive procedures. https://www.hssib.org.uk/patient-safety-investigations/retained-surgical-swabs/investigation-report/
(cited March 2026).

## Misplaced catheter

### Case 331

A 35-year-old man with a neuropathic bladder secondary to spina bifida had undergone bladder augmentation and insertion of an artificial urinary sphincter some years previously. When he attended a routine clinic appointment, he reported increasing difficulties passing intermittent catheters per urethra, as well as recent urinary tract infections. The patient was symptomatic for a urinary tract infection at the time and was admitted from clinic. A specialist nurse passed a Foley catheter as instructed by the consultant. Cloudy urine was seen to drain from the catheter at the time insertion. The patient pushed to go home that day with the indwelling catheter and oral antibiotics but returned later that night with abdominal pain and no further drainage from the catheter. Flushing the catheter resulted in drainage of some faecal debris, at which point it was suspected that the catheter was not in the bladder.

The patient was taken to theatre urgently and cystoscopy was attempted but not possible as the artificial sphincter had eroded into the urethra. Suprapubic access was therefore obtained. Subsequent computed tomography showed that the sphincter had eroded through the urethra and into the rectal wall. The patient returned to theatre for formation of a stoma and the sphincter was removed.

### Reporter’s comments

Although some urine was obtained at the time of catheter insertion, there was no further drainage. The catheter was probably in the rectum at that time. This should have been picked up before the patient went home as there was a high chance of the patient having deteriorated from sepsis and a risk of perforation of the augmented bladder due to a lack of urinary drainage.

### CORESS comments

This is an example of confirmation bias as the small amount of drainage gave false reassurance. The underlying problem (erosion of a longstanding implanted device) is an established risk of an artificial urinary sphincter. Consider asking the patient to pass the catheter themselves as their experience of self-catheterisation means they are more likely to recognise incorrect positioning. Establishing ongoing free drainage is critical.

Device erosion has been reported in relation to many implanted devices, including gastric bands and ventriculoperitoneal shunts. This complication should always be considered in a patient presenting with symptoms in the presence of such a device and history taking is therefore key.

## Missed diagnosis: caecal volvulus

### Case 332a

A 66-year-old patient presented with acute onset abdominal pain, vomiting and diarrhoea. The case was referred from the emergency department to rule out obstruction as the plain abdominal x-ray had shown a discrete distended loop of bowel in the right upper quadrant with a diameter of 11 cm. The rest of the abdomen was relatively featureless. Blood tests were within the normal range other than a mildly raised white cell count. Clinical examination of the abdomen was unremarkable: it was described as soft, non-tender and non-distended. The patient was settled and comfortable, having received analgesia in the emergency department. Safety netting advice was given, and the patient was discharged with an assumptive diagnosis of gastroenteritis.

The patient returned some hours later with worsening pain and the night on-call team re-assessed the patient. Contrast-enhanced computed tomography (CT) of the abdomen and pelvis was conducted in view of progressive symptoms. This revealed a caecal volvulus. The patient underwent a laparotomy the following morning. A type 3 caecal volvulus (caecal bascule) was found intraoperatively, the caecum was excised and an ileocolic anastomosis was carried out. The patient recovered uneventfully and was discharged on postoperative day 7.

### Reporter’s comments

The medical records (including previous abdominal scans and endoscopies) were reviewed. On previous CT of the abdomen, the patient was noted to have a high riding caecum. There should be an index of suspicion for caecal volvulus as the x-ray features revealed a featureless pattern except for the distended caecum in the right upper quadrant (in a patient known to have a high riding caecum). Although abdominal examination was benign and the patient reported a misleading symptom (diarrhoea), it is important to appreciate the value of a plain abdominal x-ray. Early CT scan should have been considered.

### CORESS comments

**Figure 1:**
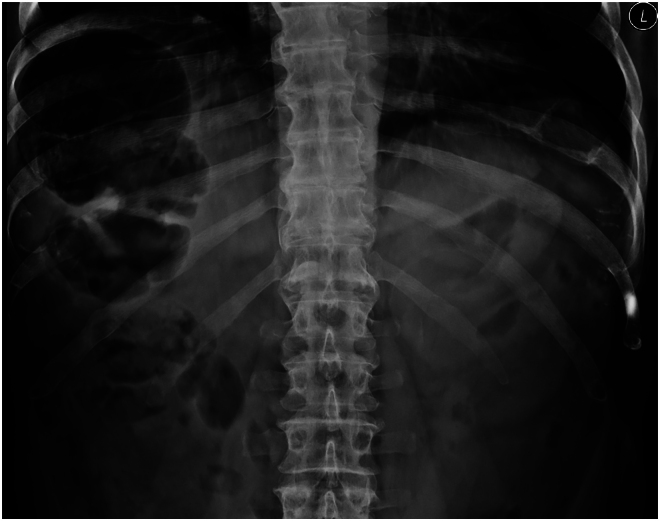
X-ray showing caecal volvulus

Caecal volvulus is an uncommon diagnosis, and the lack of symptoms and signs at the time of surgical review in the patient were reassuring. This case shows the value of good safety netting in that the patient re-presented promptly and there was no harm. The x-ray for this case is included (with permission) as pattern recognition is key (Figure 1).

## Implant misplacement

### Case 332b

A 35-year-old woman sustained multiple femoral fractures in a road traffic incident. Deep vein thrombosis prophylaxis was commenced on day 2. On day 3, just prior to planned orthopaedic surgery, she developed chest pain and dyspnoea. Angiography confirmed a pulmonary embolism. She was haemodynamically stable and her respiratory symptoms stabilised once she was anticoagulated with enoxaparin. The patient was due to undergo placement of a distal femoral plate to stabilise the fractures. The surgery was postponed for five days and the decision was then made to implant a temporary inferior vena cava filter (IVF). IVF implantation was performed and described as uneventful. The orthopaedic surgery was undertaken on the same day.

The following day, the patient complained of persistent low back pain despite analgesia. Abdominal computed tomography revealed that the proximal portion of the IVF was in an extravascular position. The patient required ongoing pain management. Minimally invasive removal of the device was not thought to be feasible.

### Reporter’s comments

The hospital safety committee reviewed the images from the IVF implantation procedure and identified an inadequate IVF release manoeuvre. The product used was new and the doctor performing the procedure noted that the new device had a different release mechanism to the previous model, which they felt was a significant factor to the inadequate positioning. Immediate training on the new device with periodic retraining was instigated.

### CORESS comments

Guidance from the National Institute for Health and Care Excellence advises commencement of mechanical venous thromboembolism (VTE) prophylaxis on admission and pharmacological VTE prophylaxis as soon as risk assessment is possible in patients with major trauma.^1^ Ideally, VTE prophylaxis should have been commenced sooner. There are few indications for placement of an IVC filter although patients with a recent proximal deep vein thrombosis or pulmonary embolism in whom anticoagulation must be interrupted to cover the operation may be considered for the procedure.^2^

Familiarity with equipment is key and training should precede use. For this procedure, it is critical for the operator to know both the device and where they are before deployment to minimise the risk of this complication.

References1.National Institute for Health and Care Excellence. Venous thromboembolism in over 16s: reducing the risk of hospital-acquired deep vein thrombosis or pulmonary embolism. https://www.nice.org.uk/guidance/ng89/
(cited March 2026).2.National Institute for Health and Care Excellence. Venous thromboembolic diseases: diagnosis, management and thrombophilia testing. https://www.nice.org.uk/guidance/ng158/
(cited March 2026).

## Implant displacement

### Case 333

A 34-year-old woman with cystic fibrosis was referred by the respiratory team for removal of an implantable port, which was blocked. The port had been in place for seven years; the port itself was on the right upper chest wall and the line entered the venous system via the internal jugular vein just above the clavicle. The patient was asymptomatic but keen for removal of the port.

During removal, the port itself was mobilised easily but the line was immovable. The surgeon opted to make an incision in the neck to try to extract the line but the line remained fixed. A decision was made to transfer the patient to the interventional radiology suite for a joint procedure, with the aim of snaring the intravascular portion of the line while the surgeon divided the line at the neck. As the interventional radiologist was setting up, the surgeon applied traction to the line, which snapped, and the intravascular portion moved quickly into the right atrium, where it could be seen moving back and forth with the contractions of the heart. Although it took some time, the interventional radiologist did manage to snare the floating portion of the line and extract it without further complication but the whole procedure took many hours, which resulted in a prolonged recovery for the patient due to her underlying condition.

### Reporter’s comments

This case highlights two issues. First, intravascular devices are known to become fixed after time and the problem might have been anticipated in this case. A plan should be made prior to surgery regarding what would be done if the line was found to be immovable. Second, during what was an unplanned joint procedure, communication between the radiologist and surgeon was poor, and the complication of a ‘line embolus’ could have been avoided.

### CORESS comments

Intravascular access devices that have been in place for several years are at risk of retained fragments at the time of removal so this scenario could have been predicted. Options for management should be discussed with the patient prior to surgery, including the possibility of port removal only.

However, when faced with an unplanned procedure with a new team, an interim team brief is advised, assuming the situation allows. Clear communication between the surgeon and radiologist could have prevented the need for line capture although it should be noted that such lines are brittle and consequently, dual snares are advised.

This article reflects the opinions of the author(s) and should not be taken to represent the policy of the Royal College of Surgeons of England unless specifically stated.
